# Transnational survey data on European farmers and consumers attitudes towards legumes adoption

**DOI:** 10.1016/j.dib.2026.112697

**Published:** 2026-03-18

**Authors:** Jerico Fiestas-Flores, Metaxia Kokkini, Anna-Lena Vollheyde, Paola Cassiano, Nikola Antonopoulos, Milan Franssen, Miriam Negussu, Rui S. Oliveira, Lucía Sanchez, Marjana Vasiljevic, Marta Goñi, Alexandros Tataridas, Helena Freitas, Federico Martinelli, Anna Camilla Moonen, Wendy Schalke, Daniel de Jong, Lara Agnioli, Ilias Travlos, Ann-Kathrin Koessler

**Affiliations:** a Institute of Environmental Planning, Leibniz University Hannover, Germany; b Laboratory of Agronomy, Department of Crop Science, Agricultural University of Athens, Athens, Greece; c Group of Agroecology, Institute of Plant Sciences, Sant'Anna School of Advanced Studies, Pisa, Italy; d DELPHY, Wageningen, the Netherlands; e Department of Biology, University of Florence, Florence, Italy; f Centre for Functional Ecology, Associate Laboratory TERRA, Department of Life Sciences, University of Coimbra, Coimbra, Portugal; g Instituto Navarro de Tecnologías e Infraestructuras Agroalimentarias (INTIA), Navarro, Spain; h Institute of Field and Vegetable Crops, Novi Sad, Serbia; i Wageningen Plant Research, Wageningen University & Research, Wageningen, the Netherlands; j School of Wine and Spirits Business, Universitè Bourgogne Europe, Burgundy School of Business, Dijon, France

**Keywords:** Protein crops, Ecosystem Services, Farmers, Consumers, Europe

## Abstract

Our article describes data from consumers’ and farmers’ online surveys about legume adoption in Germany, Greece, Italy, The Netherlands, Portugal, Serbia and Spain. Both surveys aimed to explore the enablers and barriers to adopting legumes, as well as the participant’s awareness about the ecosystem services provided by these crops. The dataset includes responses from 600 consumers and 469 farmers, obtained between June and October 2025. In addition to respondents’ attitudes towards their current and future adoption of legumes in their diets or farms, the dataset contains socio-demographic data and information about the participants’ views on the cultural ecosystem services provided by legumes. The variables allow the data to be used for analysis of consumers’ and farmers’ current preferences for legume crops in seven European countries.

Specifications Table

**Table:** 

Subject	Social Sciences
Specific subject area	Online surveys concerning European consumers and farmers’ awareness and attitudes towards legume adoption
Type of data	Table, Figure etc. Raw, Filtered, Processed etc.
Data collection	Data was collected through an online survey in SoSci Survey released in Germany, Greece, Italy, The Netherlands, Portugal, Serbia and Spain (all translated from an English baseline) between June and October 2025. The instruments’ items were derived from previous literature and included legumes current adoption, enablers, barriers, awarenes about Ecosystem Services and beliefs about legumes’ Cultural Ecosystem Services, as well as socio demographic characteristics. A total of 1069 responses (600 consumers and 469 farmers) were obtained by the survey.
Data source location	Collection: Germany, Greece, Italy, The Netherlands, Portugal, Serbia and Spain Storage country: Germany.
Data accessibility	Repository name: Zenodo Data identification number: 10.5281/zenodo.18350399) Direct URL to data: https://zenodo.org/records/18350399 Instructions for accessing these data: To access open ended answers please complete the Data Use Agreement in the supplementary materials and send it to fiestas@umwelt.uni-hannover. For peer review only: We have created a private repository to access with a password that will maintain anonymity URL: https://osf.io/tzc9e/overview?view_only=3c99647b94884f829fd0df5445fa49cb
Related research article	None

## 1 Value of the Data

• The dataset provides valuable insights about the relationships of consumers and farmers with different legume crops in seven European countries - Germany, Greece, Italy, Portugal, Serbia, Spain and the Netherlands-, including current adoption patterns, enablers, barriers and awareness about the ecosystem services provided by legumes.
• Consumer researchers can use the dataset to explore relationships between the stage of adoption (i.e., number of species, frequency of consumption) and the associated enablers and barriers in order to create tailored interventions (i.e. presentation, messages, access) to increase legume consumption in Europe.
• Agricultrual researchers can analyze how identity and sociodemographic variables influences adoption of legumes, as well as the enablers and barriers reported by European farmers. The resulting insights can inform efforts to that incentivize European farmers to plant legumes.
• The dataset includes open-text answers about the perceived benefits legumes provide to nature and humans, which could yield valuable insights into how stakeholders perceive legume crops.
• The dataset can be used by different actors at the European and national levels to understand attitudes towards legumes and the species preferred in each country.

## 2 Background

The data was collected in the context of the Horizon Europe project VALERECO – Valorization Legumes Related Ecosystem Services (ES). Legumes are an efficient source of protein [1] that also provide several ES during the production process. Nitrogen fixation and soil health tend to be ES most associated with legumes, but the crops also provide additional ecosystem services such as biodiversity conservation, water management, pollination, pest control and carbon sequestration [2,3]. Despite these benefits, the market penetration of legumes is limited [4]. It is estimated that only about 2% of arable land is used for legumes, as farmers find other crops to be more economically appealing [5]. For European consumers, legumes are preferred by a minority interested in sustainability whose primary consumption drivers are health, taste, and easy preparation. However, digestive issues, long preparation times, and lack of recipe knowledge continue to limit consumption in the region [6,7]. To increase understanding of current legume adoption patterns among European consumers and farmers, we designed and implemented online surveys across seven European countries. The instruments also collected information about awareness of ES provided by legumes, views on cultural Ecosystem Services (CES) and demographics.

## 3 Data Description

Within the Zenodo repository (https://zenodo.org/records/18350399), are four datasets and six files. These are presented in Table 1.

**Table 1:** Summary of files in the repository.

File name	Short description
(1) VALERECO_survey_data_consumers.csv	Raw data from consumers survey (in English)
(2) VALERECO_survey_data_farmers.csv	Raw data from farmers survey (in English)
(3) VALERECO_datalabels_data_consumers.csv	Data labels of raw data contained in (1)
(4) VALERECO_datalabels_data_farmers.csv	Data labels of raw data contained in (2)
(5) VALERECO_codebook_data_consumers. xlsx	Codebook of raw data contained in (1)
(6) VALERECO_codebook_data_farmers.xlsx	Codebook of raw data contained in (2)
(7) VALERECO_survey_instrument_consumers.pdf	Consumer questionnaire, including consent form (in English)
(8) VALERECO_survey_instrument_farmers.pdf	Farmer questionnaire, including consent form
(9) VALERECO_translations_consumers.zip	All the questions contained in (1) in all translated languages
(10) VALERECO_translations_farmers.zip	All the questions contained in (2) in all translated languages

The two datasets “(1) VALERECO_survey_data_consumers.csv” and “(2) VALERECO_survey_data_farmers.csv” contain all of the raw data in English for the consumer survey and the farmer survey, respectively. Open text answers are not contained in the public database and are available upon request (via email) from the corresponding author. The files “(3) VALERECO_datalabels_data_consumers.csv” and “(4) VALERECO_datalabels_data_farmers.csv” provide the full data labels for the raw data for consumers and farmers, respectively. The codebook for the consumer survey can be found in “(5) VALERECO_codebook_data_consumers.xlsx”, while the codebook for the farmer survey is in “(6) VALERECO_codebook_data_farmers.xlsx”

The files “(7) VALERECO_survey_instrument_consumers.pdf” and “(8) VALERECO_survey_ instrument_farmers.pdf” contain the questionnaires and consent forms (in English) for each group. The translations of all the consumer survey questions in all six languages are included in the document “(9) VALERECO_translations_consumers.zip”, and all of the translated farmer survey questions are in file “(10) VALERECO_translations_farmers.zip”.

Table 2 describes the general characteristics for consumers, and Table 3 presents the same information for farmers.

**Table 2:** Descriptive overview of consumer sample (*N* = 600).

Consumers	Germany	Greece	Italy	The Netherlands	Portugal	Spain	Serbia
	*N* = 67	*N* = 116	*N* = 193	*N* = 67	*N* = 48	*N* = 58	*N* = 51
**Average age (years)**	26.75	35.07	39.10	40.42	42.58	52.80	41.41
**Sex**							
Female	67 %	59 %	62 %	60 %	56 %	69 %	90 %
Male	33 %	39 %	35 %	34 %	44 %	31 %	10 %
Other	0 %	0 %	1 %	0 %	0 %	0 %	0 %
Prefer not to say	0 %	2 %	2 %	6 %	0 %	0 %	0 %
**Education level**							
Primary education	0 %	2 %	0 %	3 %	2 %	3 %	0 %
Lower secondary education	0 %	0 %	1 %	4 %	0 %	2 %	0 %
Upper secondary education (e.g., high school)	49 %	17 %	11 %	3 %	29 %	9 %	18 %
Post-secondary nontertiary (e.g., pre-vocational)	1 %	5 %	3 %	4 %	0 %	2 %	0 %
Short-cycle tertiary (e.g., associate’s degree)	3 %	3 %	1 %	7 %	4 %	12 %	2 %
Bachelor’s or equivalent	19 %	37 %	17 %	28 %	23 %	38 %	33 %
Master‘s or equivalent	18 %	31 %	50 %	43 %	31 %	12 %	33 %
Doctoral or equivalent	7 %	3 %	16 %	4 %	10 %	21 %	12 %
Other (Please specify)	1 %	0 %	2 %	0 %	0 %	2 %	2 %
Prefer not to answer	0 %	3 %	1 %	1 %	0 %	0 %	0 %
**Wage percentile**							
1st percentile	43 %	45 %	46 %	20 %	46 %	0 %	34 %
2nd percentile	36 %	11 %	28 %	25 %	46 %	27 %	0 %
3rd percentile	36 %	33 %	17 %	28 %	46 %	27 %	34 %
4th percentile	17 %	23 %	46 %	43 %	15 %	17 %	0 %
5th percentile	17 %	46 %	42 %	28 %	25 %	23 %	36 %
6th percentile	36 %	44 %	36 %	18 %	13 %	33 %	31 %
7th percentile	36 %	18 %	15 %	18 %	19 %	13 %	31 %
8th percentile	37 %	18 %	17 %	25 %	15 %	13 %	16 %
9th percentile	18 %	13 %	45 %	46 %	15 %	17 %	18 %
10th percentile	17 %	22 %	44 %	46 %	45 %	46 %	20 %
I don't know	17 %	11 %	25 %	28 %	46 %	27 %	34 %
Prefer not to answer	17 %	15 %	17 %	19 %	42 %	26 %	18 %

Note: The variables used from the dataset to build this table are residency (C103), age (C507_01), gender (C508), education level (C509) and income (C510, C514-C519). Missing answers were excluded.

**Table 3:** Descriptive overview of farmer sample (*N* = 469).

Farmers	Germany	Greece	Italy	The Netherlands	Portugal	Spain	Serbia
	*N* = 9	*N* = 100	*N* = 139	*N* = 75	*N* = 47	*N* = 49	*N* = 50
**Average age (years)**	47	42.76	48.10	50.27	48.33	47.62	48.38
**Sex**							
Female	11 %	36 %	24 %	22 %	28 %	26 %	8 %
Male	78 %	59 %	74 %	77 %	68 %	72 %	90 %
Other	0 %	0 %	0 %	0 %	2 %	0 %	2 %
Prefer not to say	11 %	5 %	1 %	1 %	2 %	2 %	0 %
**Education level**							
Primary education	0 %	0 %	1 %	3 %	0 %	2 %	6 %
Lower secondary education	0 %	2 %	8 %	3 %	17 %	2 %	10 %
Upper secondary education (e.g., high school)	11 %	21 %	23 %	8 %	36 %	46 %	12 %
Post-secondary nontertiary (e.g., pre-vocational)	11 %	7 %	3 %	15 %	15 %	0 %	14 %
Short-cycle tertiary (e.g., associate’s degree)	11 %	6 %	0 %	9 %	0 %	0 %	18 %
Bachelor’s or equivalent	11 %	44 %	19 %	35 %	19 %	26 %	29 %
Master‘s or equivalent	22 %	15 %	36 %	26 %	6 %	8 %	10 %
Doctoral or equivalent	11 %	2 %	5 %	1 %	2 %	10 %	0 %
Other (Please specify)	11 %	0 %	4 %	0 %	0 %	2 %	0 %
Prefer not to answer	11 %	3 %	1 %	0 %	4 %	4 %	0 %
**Wage percentile**							
1st percentile	22 %	1 %	4 %	3 %	2 %	4 %	0 %
2nd percentile	0 %	3 %	11 %	8 %	2 %	2 %	2 %
3rd percentile	0 %	4 %	11 %	8 %	2 %	4 %	0 %
4th percentile	11 %	5 %	8 %	14 %	9 %	4 %	6 %
5th percentile	0 %	7 %	9 %	15 %	26 %	2 %	6 %
6th percentile	0 %	19 %	7 %	11 %	15 %	6 %	6 %
7th percentile	0 %	12 %	6 %	5 %	11 %	6 %	2 %
8th percentile	33 %	11 %	9 %	1 %	9 %	6 %	10 %
9th percentile	0 %	10 %	8 %	0 %	6 %	2 %	4 %
10th percentile	11 %	1 %	10 %	1 %	9 %	10 %	2 %
I don't know	11 %	2 %	2 %	4 %	0 %	18 %	0 %
Prefer not to answer	11 %	25 %	15 %	30 %	11 %	36 %	61 %

Note: The variables used from the dataset to build this table are residency (F005), age (F607_01), gender (F608), education level (F609) and income (F520-F526). Missing answers were excluded.

**Fig. 1:**
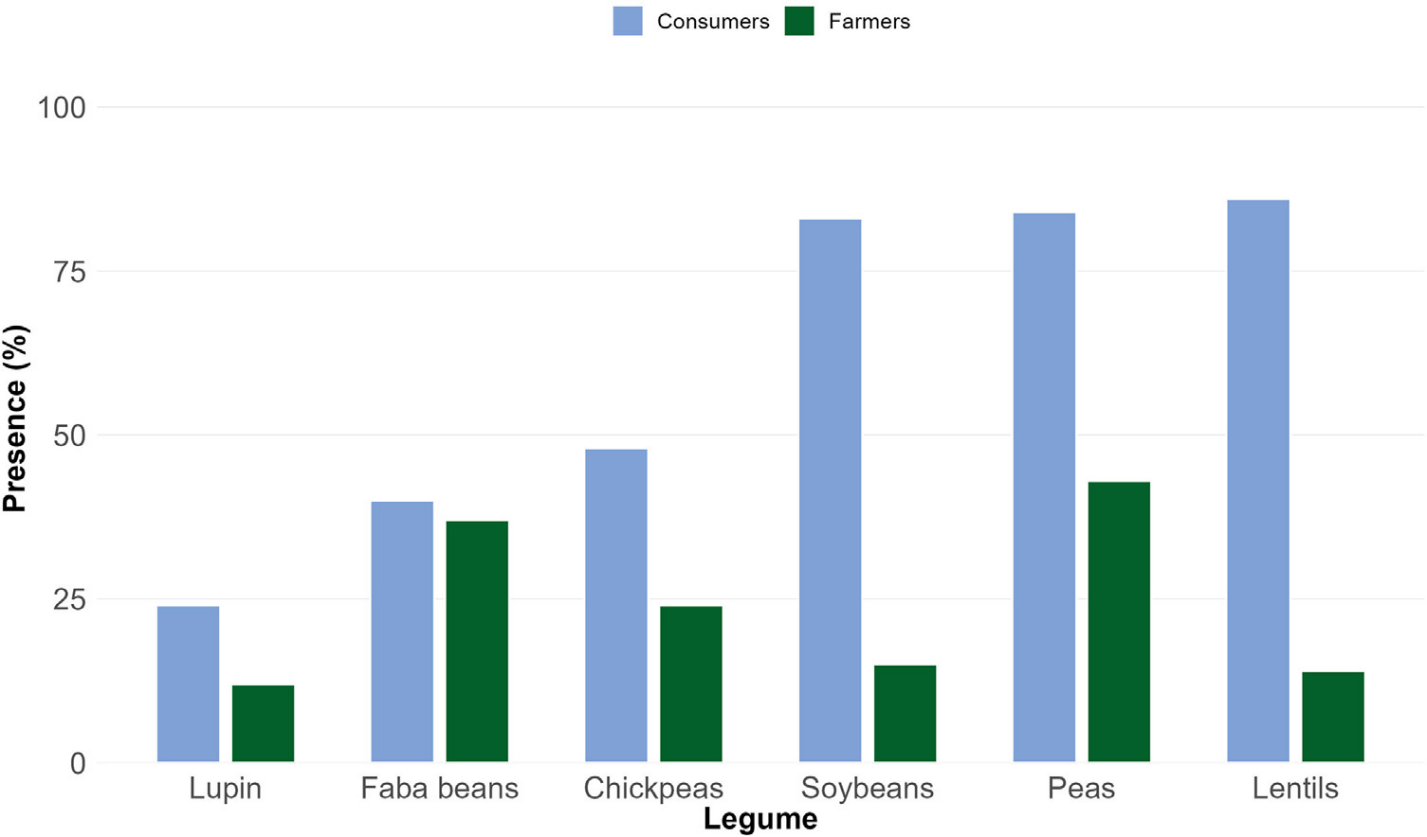
Presence of legumes in consumers’ diets and on farmers’ lands in 2024 by legume type.

The data presented in this article can be utilized in different ways to analyze consumers’ or farmers’ awareness, behavior, and attitudes toward legumes. For instance, the data allow us to analyze the presence of different legumes in participants’ diets last month or on farms in 2024 (see Fig. 1). The consumer dataset also contains information about the frequency and form of legume consumption. The farmers dataset also includes information about on-farm legume production.

**Fig. 2:**
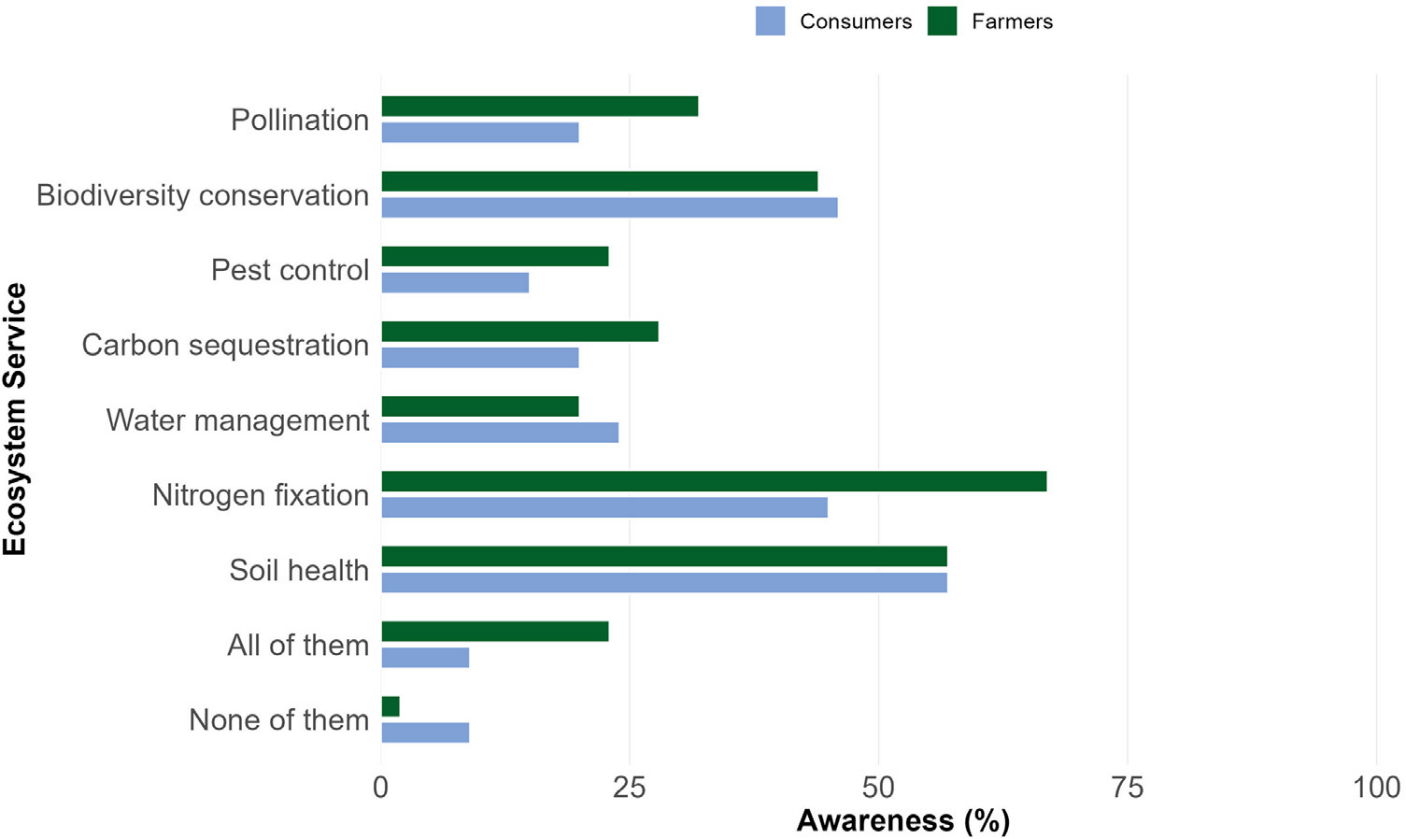
ES provided by legumes as identified by consumers and farmers.

Note: The variables used in this figure are consumers’ ES responses (C409_01-C409_07, C409_10-C409_11) and farmers’ CES responses (F513-F522). Missing answers were excluded.

Similarly, the data set contains information about awareness of the ES provided by legumes. We first asked participants how they think legumes affect humans and nature (open-text format) and then asked them to select which ES they had associated with legumes prior the survey. Participants had to choose from a pre-established list with multiple response options, including the following ES: pollination, biodiversity conservation, pest control, carbon sequestration, water management, nitrogen fixation and soil health. Fig 2. presents a descriptive summary with each ES and the corresponding awareness percentage for each group.

**Fig. 3:**
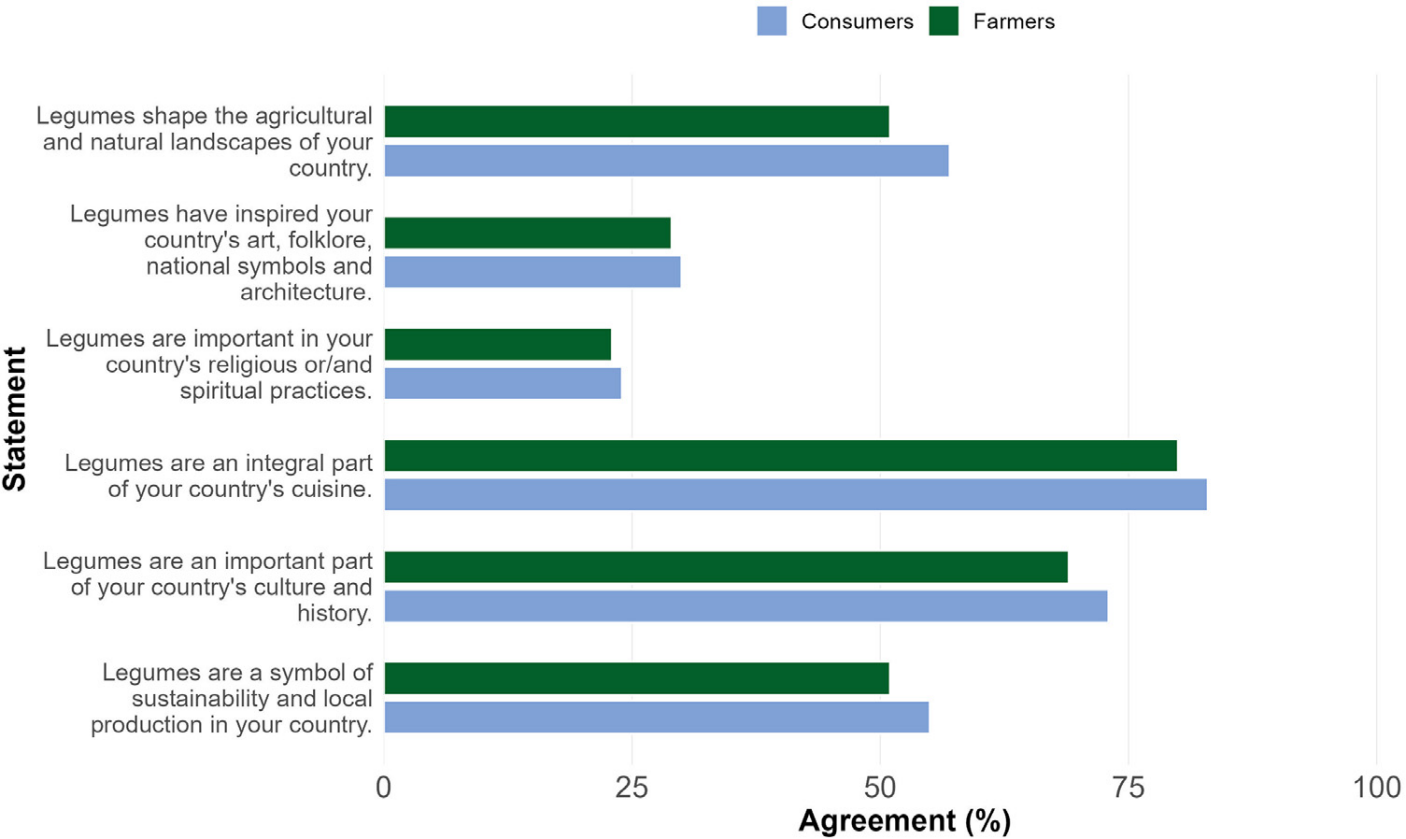
Consumers and farmers agreeing or strongly agreeing with statements about legumes and CES.

Note: The variables used in this figure are consumers’ CES responses (C403_01-C403_06) and farmers’ CES responses (F503_01-F503_06). Missing answers were excluded.

Additionally, the dataset can sheds light on the importance of legumes as a provider of different CES. In the survey each participant had to assign values from 1 to 5 for different statements linking legumes with CES as follows: 1: Strongly disagree, 2: Disagree, 3: Neither agree or disagree, 4: Agree, 5: Strongly agree. Fig. 3 displays the percentage of participants who agreed or strongly agreed with each statement.

Furthermore, the dataset provides information about enablers and barriers to future legume adoption as identified by consumers and farmers. Consumers were asked to indicate the reasons for consuming each legume from a list of nine items identified from the literature, while farmers were asked to select their motivations for planting legumes from a list of 15 items. A similar process was implemented for the barriers: consumers were asked to select the main barriers to increasing their consumption (14 items) and farmers were asked to indicate the main limitations for planting legume crops on their land (15 items). Table 4 shows a descriptive summary of each group’s three most relevant reasons and limitations for adopting legumes. Consumers cited the taste and nutritional properties of legumes as the main drivers for consumption, while 43% of consumers indicated that they had no particular reason not eat legumes. Farmers’ motivations were predominantly related to the benefits for soil health and nitrogen availability, while the main barriers to expanding legume production were low profit margins, yield instability, and market access.

**Table 4:** The three most important enablers and barriers to adopt legumes for consumers and farmers.

**Enablers**			
Item	Consumers	Item	Farmers
I like the taste	61 %	Improves soil fertility and soil health.	61 %
Nutritional properties	46 %	Increases nitrogen availability.	53 %
Easy to prepare	35 %	I want to diversify my crop rotation.	52 %

**Barriers**			

Item	Consumers	Item	Farmers

I have no reason to limit consumption	43 %	Low profit margins	34 %
Eating habits	26 %	Yield instability	31 %
Hard to digest	25 %	Limited/ lack of market access and demand	26 %

Note: The variables in this table are consumers’ enablers (C209-C214), consumers’ barriers (C219, C302), farmers’ enablers (F302_01-F302_15) and farmers’ barriers (F404, F507). Missing answers were excluded.

The previous descriptive tables and figures provide an overview of what can be explored in the dataset. To exploit the uniqueness of the dataset, the authors intend to write and submit a paper that analyzes enablers and barriers based on the consumers’ stage of adoption and the farmers’ percentage of land allocated to legumes. The dataset enables crossing of these variables and the use of parametric analyzes to identify statistically significant differences between actors and their current level of legume adoption. This forthcoming analysis will not overlap with the current publication, which is descriptive in nature and does not use any model to identify associations between variables.

## 4 Experimental Design, Materials and Methods

We developed one only survey for consumers and one for farmers (all over the age of 18) in Germany, Greece, Italy, the Netherlands, Portugal, Serbia, and Spain. Participants were recruited by research institutions working in VALERECO and Living Labs (LL) using a non-probabilistic convenience sampling method, with the questionnaire being distributed online via email, messaging applications (i.e., WhatsApp), and social media (i.e., LinkedIn, Facebook). The LL partners also promoted the survey by displaying at universities, local markets, farmers associations and convenient stores. To incentivize participation, all communications indicated that participants who completed the survey could enter a lottery to win 100 EUR (consumers) or 150 EUR (farmers). To enter, participants had to provide an e-mail address, which could not be linked to their responses to preserve anonymity.

The consumer survey consisted of three main sections. The first was about their consumption patterns for different food groups, including legumes, during the last month. Participants who indicated that they had consumed legumes were presented with follow-up questions about frequency, presentation, enablers, and barriers. Those who reported that they had not consumed legumes were presented with questions related to barriers and changes that would be necessary to increase their consumption in the future. Before moving to the next section, both groups were asked if they intend to consume more legumes in the future. Section two consisted of questions about the relationship of legumes and ES, including questions about CES. In this section, participants also received information about the ES provided by legumes according to the literature, and they were then presented with the same questions about their potential future legume consumption. The last section collected participant’s demographic information including age, gender, education level, income and dietary preferences.

The farmer survey had a similar structure and objectives, but some questions were different as the focus was to understand the adoption of legumes in farming. The first section of the instrument asked about the farmer’s story using a narrative identity approach [8] and about their farm’s characteristics. Those who reported growing legume were presented with follow-up questions about their motivations and barriers to cultivating more legumes on their land, as well as specific information about how they used legumes. Farmers who stated that they did not grow legumes were presented with questions about their reasons. As in the consumer survey, the farmers were asked if they intend to use more legumes in the future. The second section asked similar questions to the consumer instrument (e.g., ES awareness, CES importance), and then the farmers read information about the ES provided by legumes before being asked again about their intention to use more legumes in the future. In the last section, we collected participant demographics, including age, gender, education, income, and a farmer identity scale [9]. In both survey versions, the demographic questions were based on those used by EuroBarometer (2024) and the income percentiles were based on the European Social Survey [10].

Although both instruments had similar sections, the consumer and farmer datasets differ in the level of detail requested for specific questions. For example, consumers were asked to indicate the reasons to consume each species of legumes they had selected previously in the questionnaire, while farmers were asked for their general motivation to grow legumes. Additionally, farmers were asked questions about their farms’ characteristics and their identity. For both instruments, the variable of interest was the level of ES awareness, which was obtained by asking the participant to indicate which of seven ES they considered to be provided by legumes. Another interest variable was the awareness of CES provided by legumes, which was addressed in both instruments using a Likert scale (1 to 5) for the participant to indicate their level of agreement with different statements linking legumes with CES (see Fig. 3). Each question was formulated on the existing literature on legume ES and ES awareness.

Both surveys were created in English and translated using DeepL pro to Dutch, German, Greek, Italian, Portuguese, Serbian, and Spanish. The initial translations were sent to selected LL partners native speakers and familiar with the legume environment and terminology to review and validate the questions for each instrument. We met over a two months period to finalize the translations to make the survey available in all eight languages. Once the farmer survey was validated by all LL, we tested it with six German farmers and VALERECO’s partners involved in agroecological research. The consumers’ survey was tested with researchers at Leibniz University Hannover who were not involved in research about legumes.

The instruments were administered to a total of 619 consumers and 491 farmers. These responses were subject to a quality control process using Rstudio (Posit [11]) that included different checks. First, the completion time for the entire survey was calculated and we eliminated responses more than two standard deviations below the median to exclude speeders and/or participants paying low attention to the questions. Second, we examined the presence of suspicious response patterns in the Likert scales and eliminated responses from participants who selected extreme-value items that contradicted each other in all the survey scales, as this pattern would indicated that the participant was not paying attention to the questionnaire. Third, we assess the use of chatbots or large language model (LLM) in open-ended responses. We calculated how many seconds answers would take to produce at a rate of 200 characters per minute and compared this benchmark time with the actual time spent by each participant on the open-ended questions page. We excluded responses when the time at the page was less than one-third of the calculated benchmark time, which indicated that the survey had likely been completed by a chatbot or LLM (Schles and McKenzie, 2025). All responses excluded in this manner were long answers generated in a short time without any typos. After excluding observations using the criteria mentioned above the final raw dataset for this article includes 600 consumers and 469 farmers.

## Limitations

Although the dataset provides valuable insights about consumers’ and farmers’ legume adoption, the main limitations of this dataset are the sampling bias caused by convenience sampling and the limited geographical coverage, as the instruments were implemented in only seven European countries. Additionally, online recruitment increased the likelihood of self-selection bias and of pre-existing familiarity or fondness with legumes. These limitations reduce the generalizability of the dataset and inferences of its results to the covered countries. Future research could use the present dataset and instruments to explore the adoption of legumes and other plant-based protein options among different populations using sampling methods that ensure population representativeness.

## Ethics Statement

Prior to survey completion, participants were required to provide informed consent. The information sheet, the consent forms, and the study design for both surveys were approved by the Ethics Committee of the Burgundy School of Business Research (CERBSB2025–87).

## CRediT Author Statement

Jerico Fiestas-Flores*: Conceptualization, project administration, methodology, software, investigation, data curation, writing, original draft, visualization; Metaxia Kokkini: Resources, project administration, investigation; Anna-Lena Vollheyde: Conceptualization, validation, resources, investigation; Paola Cassiano: Resources, investigation; Nikola Antonopoulos: Resources, investigation; Milan Franssen: Investigation; Miriam Negussu: Resources, investigation; Rui S. Oliveira: Resources, investigation; Lucía Sanchez: Resources, investigation; Marjana Vasiljevic: Resources, investigation; Marta Goñi: Resources, investigation; Alexandros Tataridas: Investigation; Helena Freitas: Investigation; Federico Martinelli: Resources, investigation; Anna Camilla Moonen: Resources, investigation; Wendy Schalke: Resources, investigation; Daniel de Jong: Resources, investigation; Lara Agnioli: Resources, supervision; Ilias Travlos (AUA): Resources, supervision; Ann-Kathrin Koessler (LUH): Conceptualization, supervision, methodology, writing, reviewing and editing

## Data Availability

ZenodoTransnational survey data on European farmers and consumers attitudes towards legumes adoption (Original data)
OSFTransnational survey data on European farmers and consumers attitudes towards legumes adoption - Reviewers (Original data)
